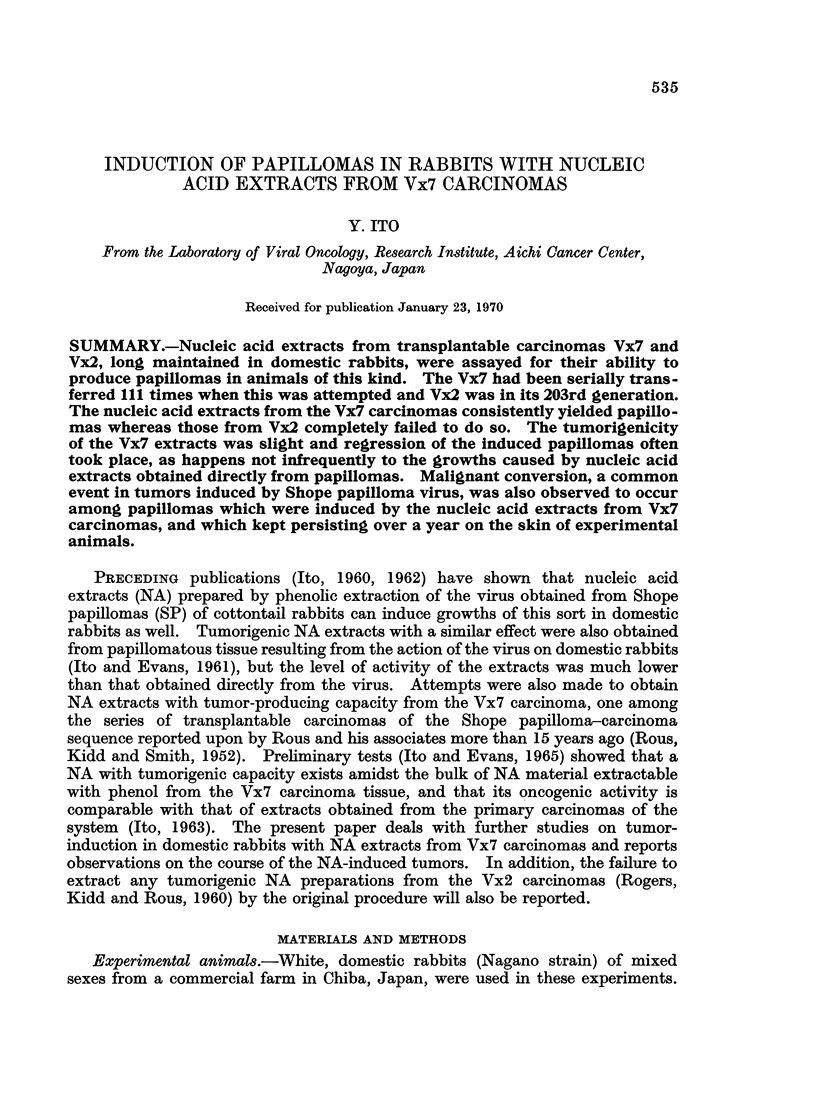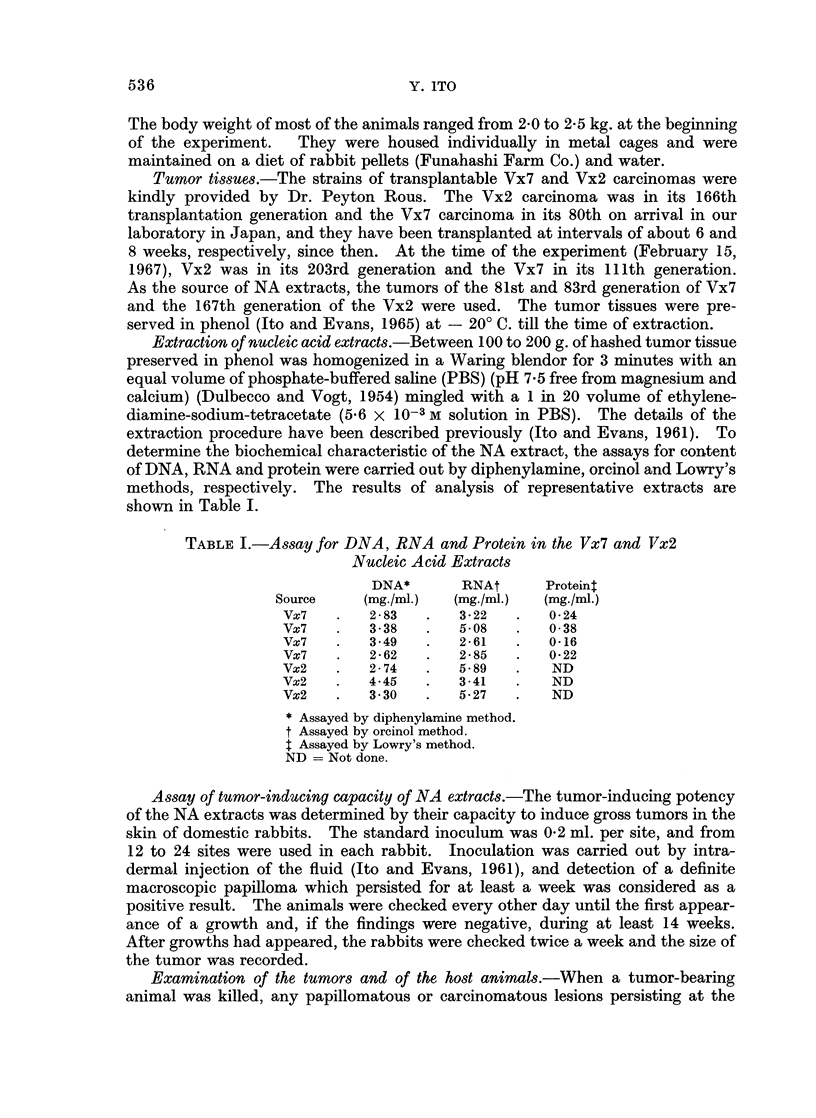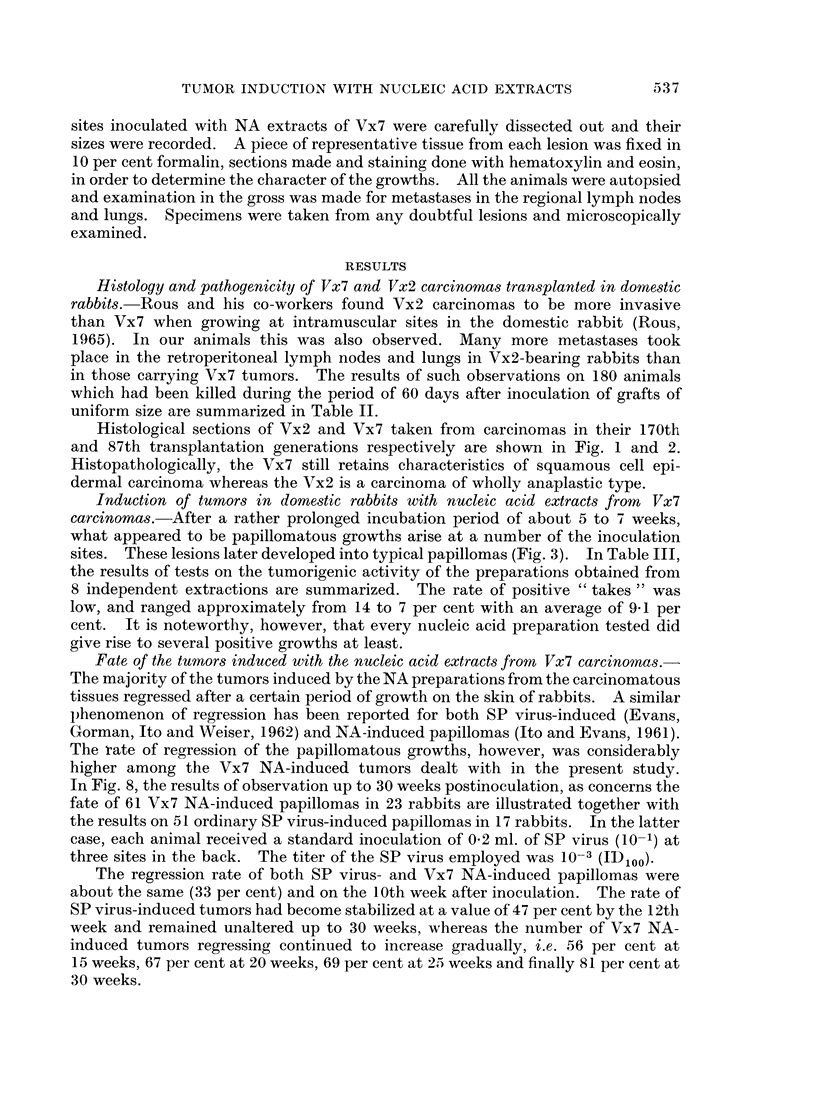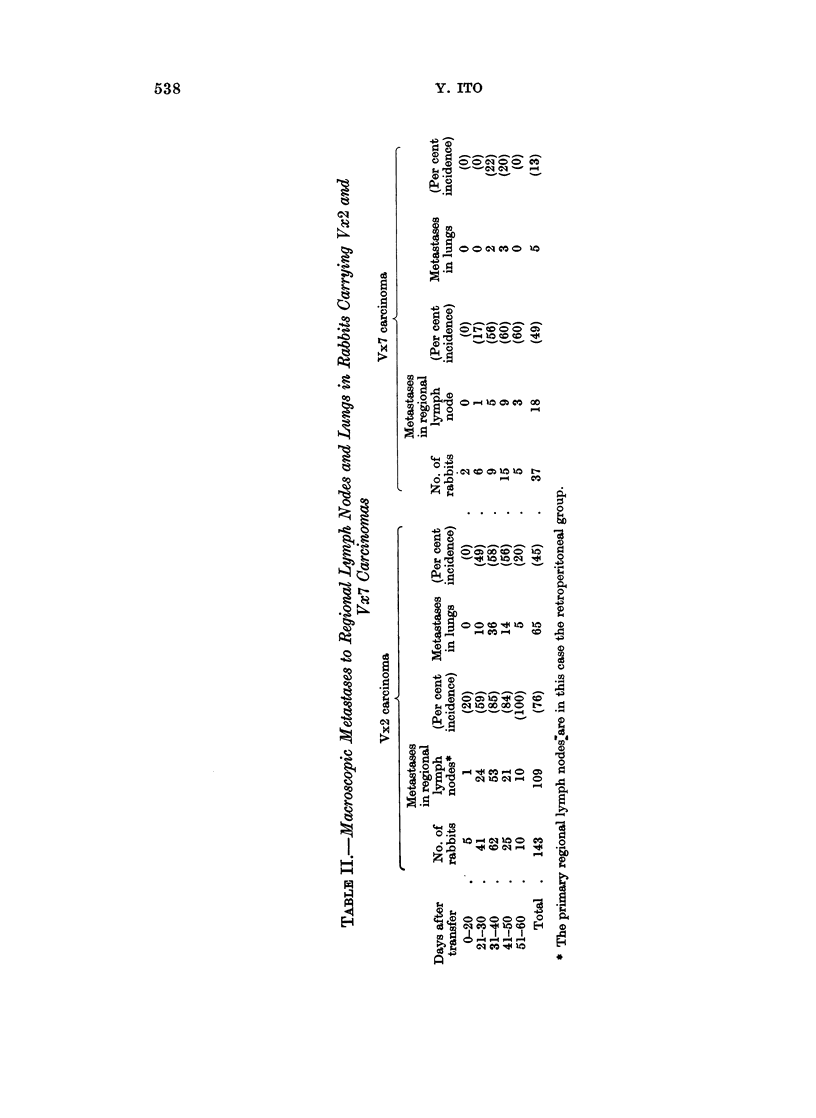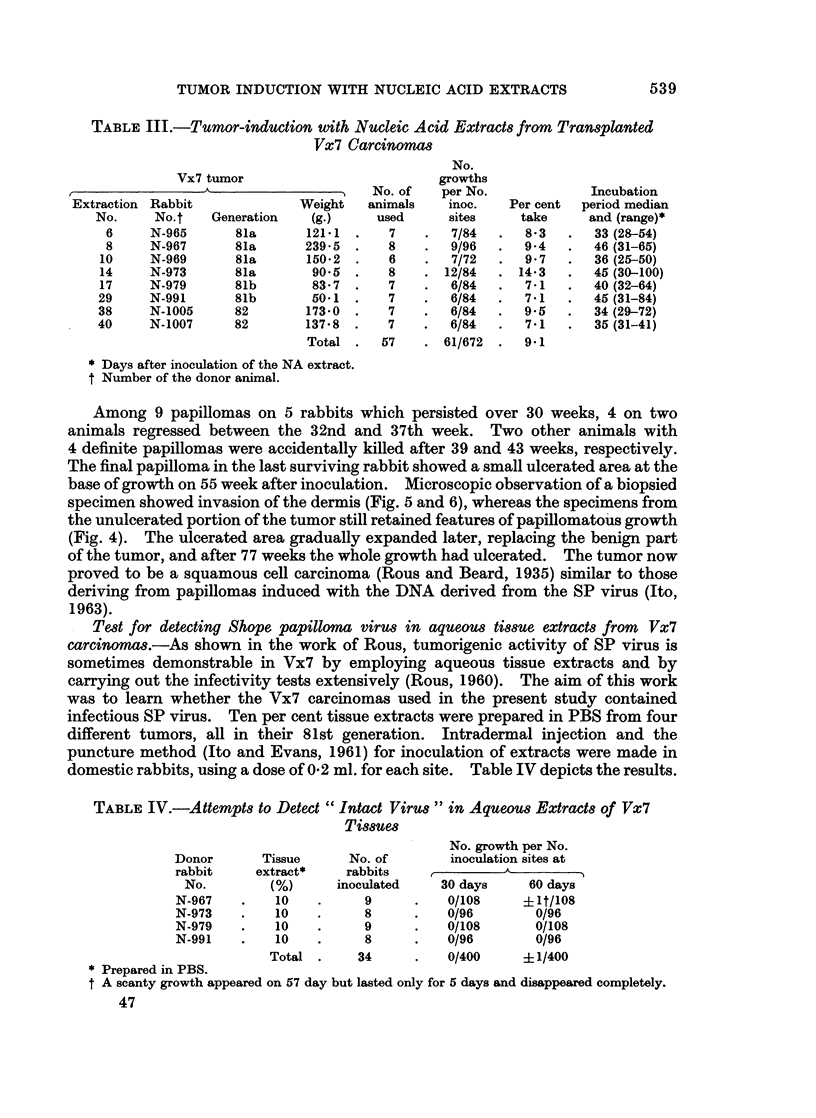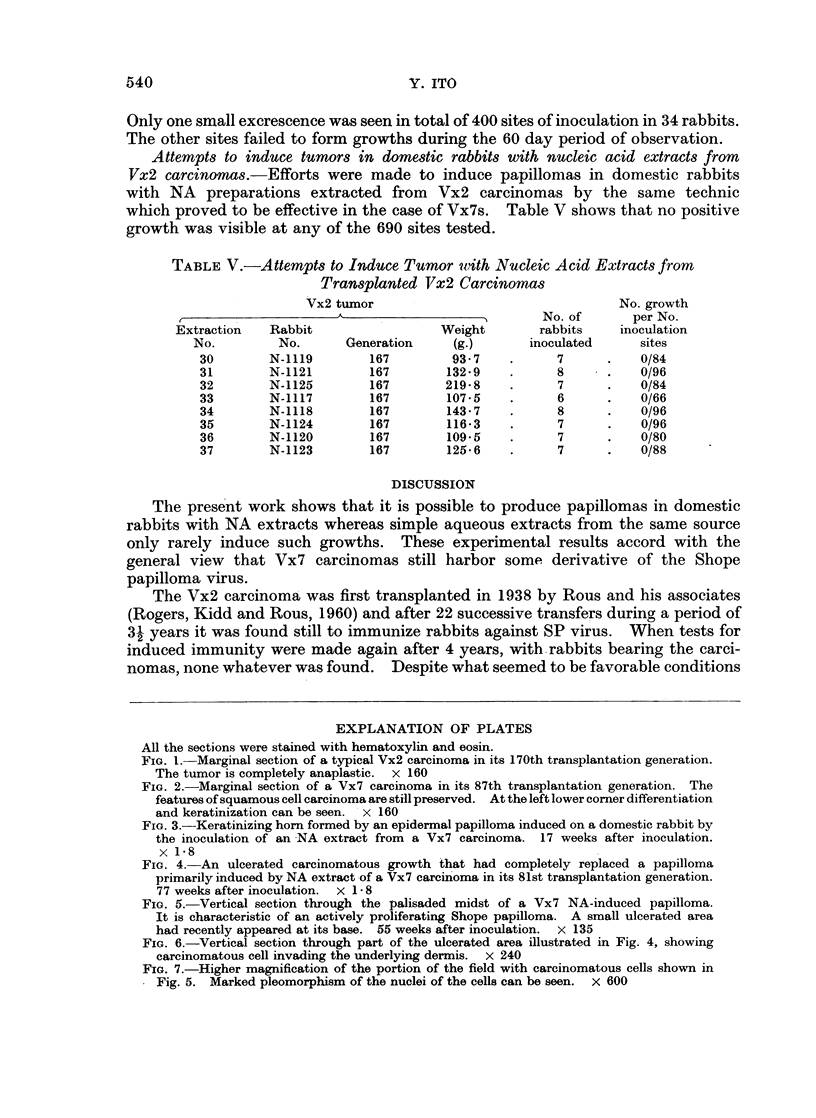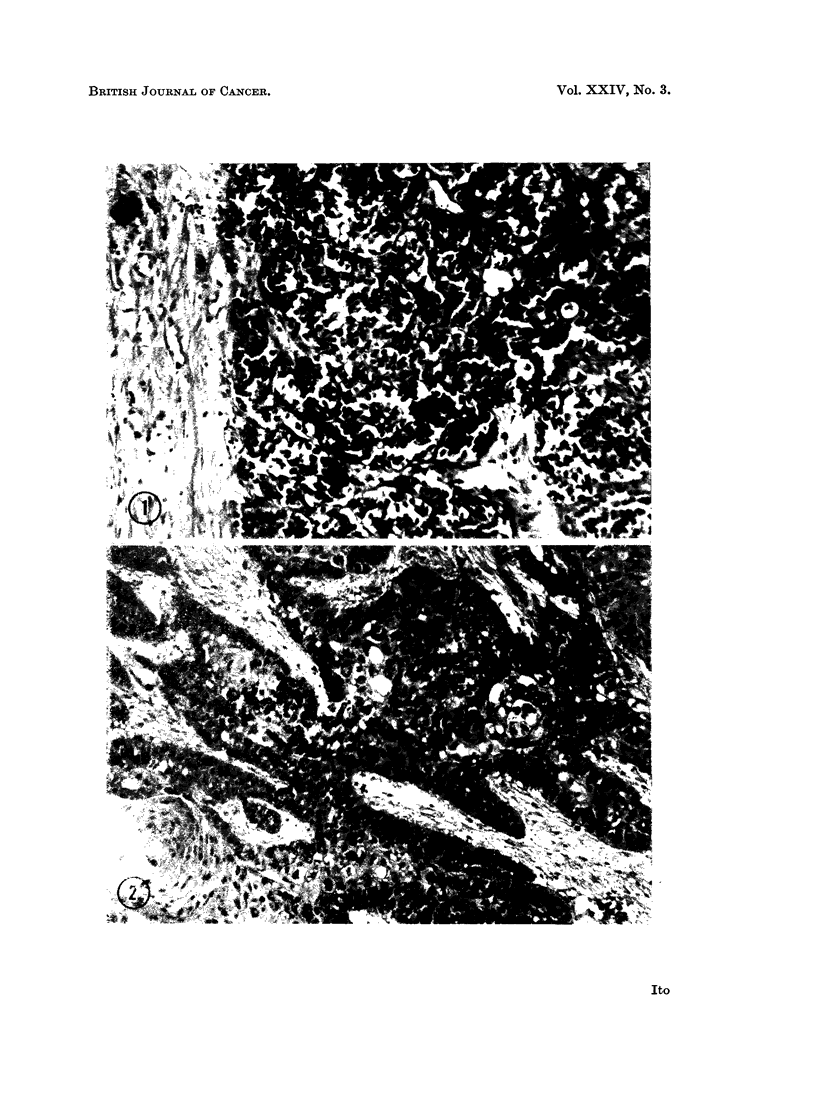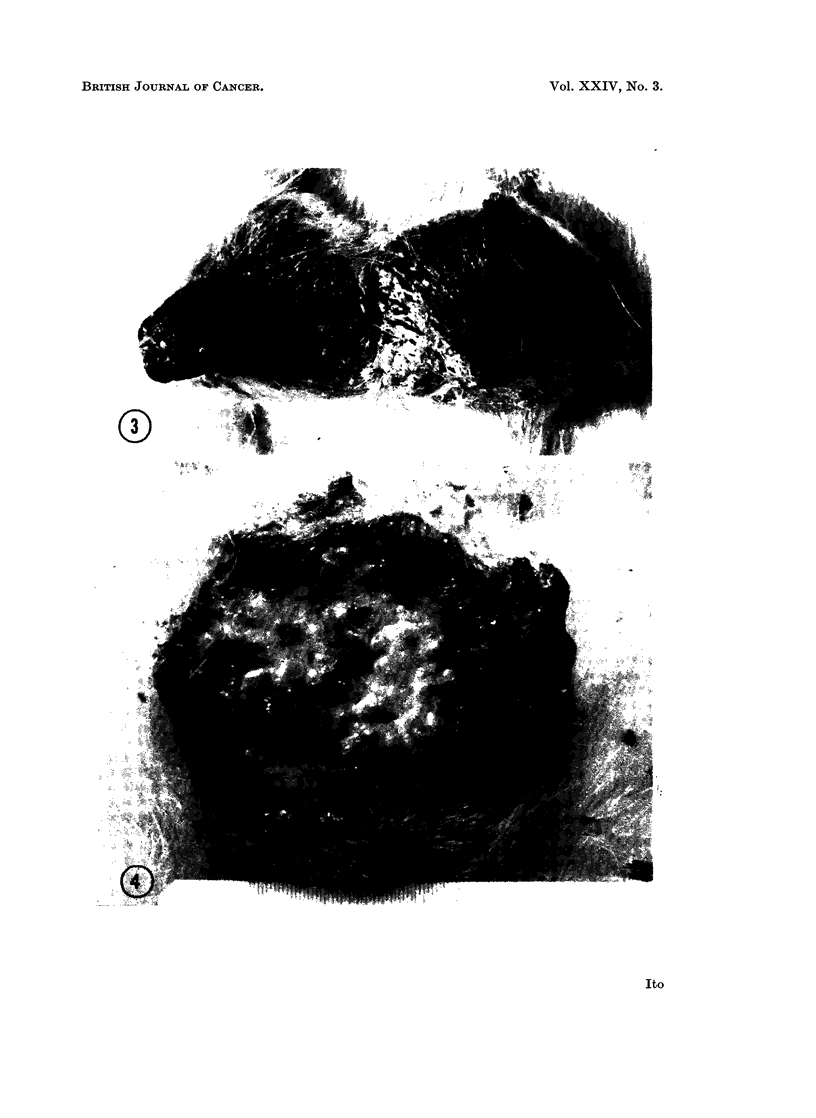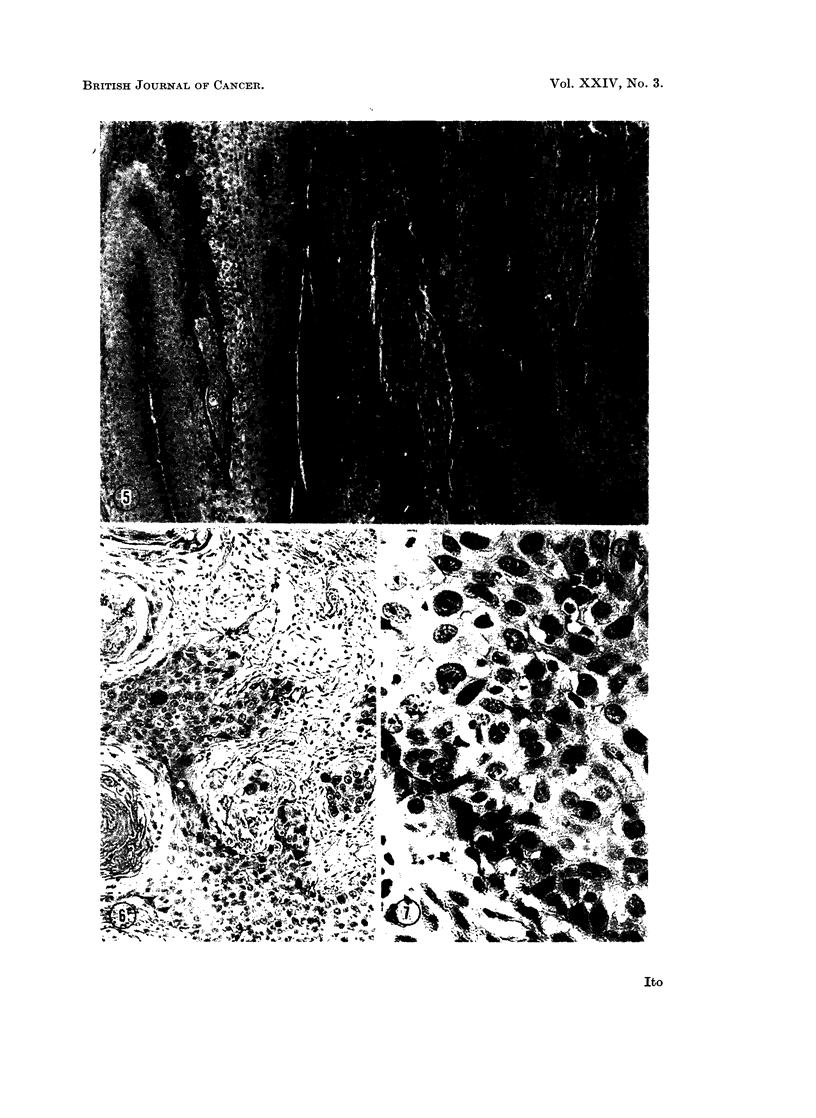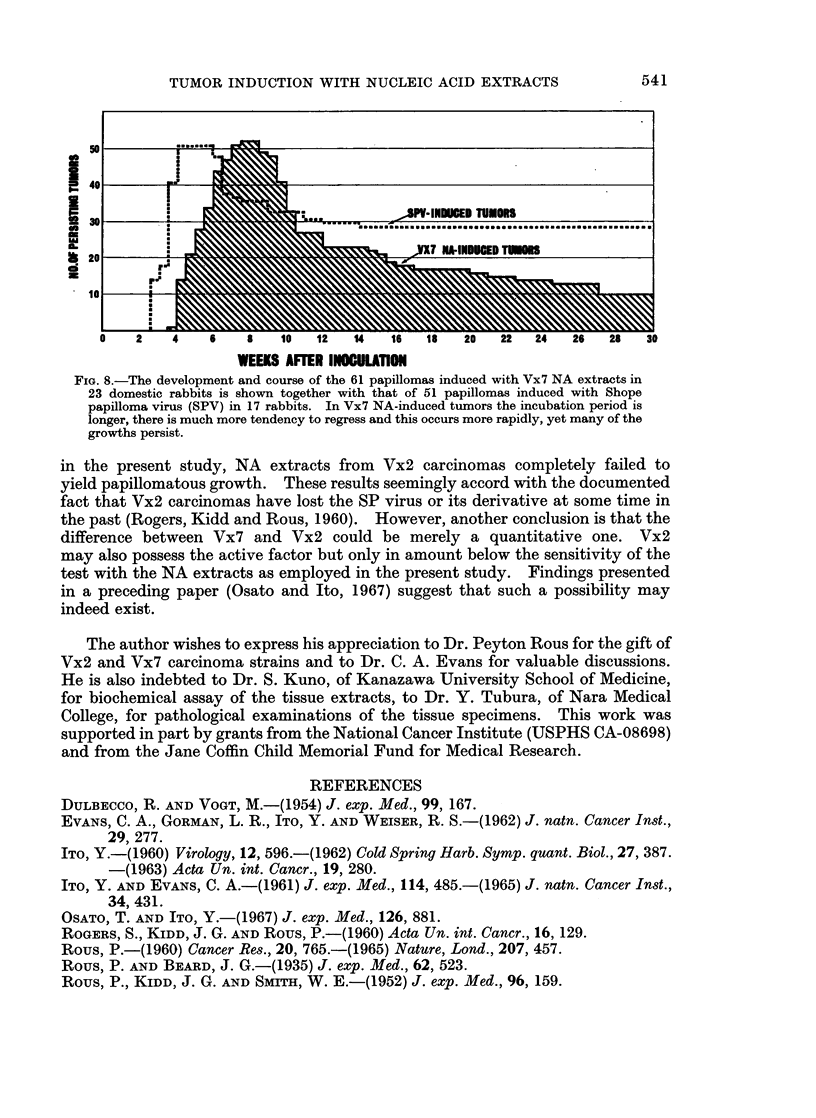# Induction of Papillomas in Rabbits with Nucleic Acid Extracts from Vx7 Carcinomas

**DOI:** 10.1038/bjc.1970.64

**Published:** 1970-09

**Authors:** Y. Ito

## Abstract

**Images:**


### INDUCTION OF PAPILLOMAS IN RABBITS WITH NUCLEIC ACID EXTRACTS FROM Vx7 CARCINOMAS

Y. ITO

*From the Laboratory of Viral Oncology, Research Institute, Aichi Cancer Center, Nagoya, Japan*

Received for publication January 23, 1970

**SUMMARY.—Nucleic acid extracts from transplantable carcinomas Vx7 and Vx2, long maintained in domestic rabbits, were assayed for their ability to produce papillomas in animals of this kind. The Vx7 had been serially transferred 111 times when this was attempted and Vx2 was in its 203rd generation. The nucleic acid extracts from the Vx7 carcinomas consistently yielded papillomas whereas those from Vx2 completely failed to do so. The tumorigenicity of the Vx7 extracts was slight and regression of the induced papillomas often took place, as happens not infrequently to the growths caused by nucleic acid extracts obtained directly from papillomas. Malignant conversion, a common event in tumors induced by Shope papilloma virus, was also observed to occur among papillomas which were induced by the nucleic acid extracts from Vx7 carcinomas, and which kept persisting over a year on the skin of experimental animals.**

PRECEDING publications (Ito, 1960, 1962) have shown that nucleic acid extracts (NA) prepared by phenolic extraction of the virus obtained from Shope papillomas (SP) of cottontail rabbits can induce growths of this sort in domestic rabbits as well. Tumorigenic NA extracts with a similar effect were also obtained from papillomatous tissue resulting from the action of the virus on domestic rabbits (Ito and Evans, 1961), but the level of activity of the extracts was much lower than that obtained directly from the virus. Attempts were also made to obtain NA extracts with tumor-producing capacity from the Vx7 carcinoma, one among the series of transplantable carcinomas of the Shope papilloma–carcinoma sequence reported upon by Rous and his associates more than 15 years ago (Rous, Kidd and Smith, 1952). Preliminary tests (Ito and Evans, 1965) showed that a NA with tumorigenic capacity exists amidst the bulk of NA material extractable with phenol from the Vx7 carcinoma tissue, and that its oncogenic activity is comparable with that of extracts obtained from the primary carcinomas of the system (Ito, 1963). The present paper deals with further studies on tumor-induction in domestic rabbits with NA extracts from Vx7 carcinomas and reports observations on the course of the NA-induced tumors. In addition, the failure to extract any tumorigenic NA preparations from the Vx2 carcinomas (Rogers, Kidd and Rous, 1960) by the original procedure will also be reported.

### MATERIALS AND METHODS

*Experimental animals.*—White, domestic rabbits (Nagano strain) of mixed sexes from a commercial farm in Chiba, Japan, were used in these experiments.

The body weight of most of the animals ranged from 2·0 to 2·5 kg. at the beginning of the experiment. They were housed individually in metal cages and were maintained on a diet of rabbit pellets (Funahashi Farm Co.) and water.

*Tumor tissues.*—The strains of transplantable Vx7 and Vx2 carcinomas were kindly provided by Dr. Peyton Rous. The Vx2 carcinoma was in its 166th transplantation generation and the Vx7 carcinoma in its 80th on arrival in our laboratory in Japan, and they have been transplanted at intervals of about 6 and 8 weeks, respectively, since then. At the time of the experiment (February 15, 1967), Vx2 was in its 203rd generation and the Vx7 in its 111th generation. As the source of NA extracts, the tumors of the 81st and 83rd generation of Vx7 and the 167th generation of the Vx2 were used. The tumor tissues were preserved in phenol (Ito and Evans, 1965) at − 20° C. till the time of extraction.

*Extraction of nucleic acid extracts.*—Between 100 to 200 g. of hashed tumor tissue preserved in phenol was homogenized in a Waring blendor for 3 minutes with an equal volume of phosphate-buffered saline (PBS) (pH 7·5 free from magnesium and calcium) (Dulbecco and Vogt, 1954) mingled with a 1 in 20 volume of ethylenediamine-sodium-tetracetate ($5{\cdot}6 \times 10^{-3}$ M solution in PBS). The details of the extraction procedure have been described previously (Ito and Evans, 1961). To determine the biochemical characteristic of the NA extract, the assays for content of DNA, RNA and protein were carried out by diphenylamine, orcinol and Lowry's methods, respectively. The results of analysis of representative extracts are shown in Table I.

TABLE I.—*Assay for DNA, RNA and Protein in the Vx7 and Vx2 Nucleic Acid Extracts*

| Source | DNA* (mg./ml.) | RNA† (mg./ml.) | Protein‡ (mg./ml.) |
|---|---|---|---|
| Vx7 | 2·83 | 3·22 | 0·24 |
| Vx7 | 3·38 | 5·08 | 0·38 |
| Vx7 | 3·49 | 2·61 | 0·16 |
| Vx7 | 2·62 | 2·85 | 0·22 |
| Vx2 | 2·74 | 5·89 | ND |
| Vx2 | 4·45 | 3·41 | ND |
| Vx2 | 3·30 | 5·27 | ND |

* Assayed by diphenylamine method.
† Assayed by orcinol method.
‡ Assayed by Lowry's method.
ND = Not done.

*Assay of tumor-inducing capacity of NA extracts.*—The tumor-inducing potency of the NA extracts was determined by their capacity to induce gross tumors in the skin of domestic rabbits. The standard inoculum was 0·2 ml. per site, and from 12 to 24 sites were used in each rabbit. Inoculation was carried out by intradermal injection of the fluid (Ito and Evans, 1961), and detection of a definite macroscopic papilloma which persisted for at least a week was considered as a positive result. The animals were checked every other day until the first appearance of a growth and, if the findings were negative, during at least 14 weeks. After growths had appeared, the rabbits were checked twice a week and the size of the tumor was recorded.

*Examination of the tumors and of the host animals.*—When a tumor-bearing animal was killed, any papillomatous or carcinomatous lesions persisting at the sites inoculated with NA extracts of Vx7 were carefully dissected out and their sizes were recorded. A piece of representative tissue from each lesion was fixed in 10 per cent formalin, sections made and staining done with hematoxylin and eosin, in order to determine the character of the growths. All the animals were autopsied and examination in the gross was made for metastases in the regional lymph nodes and lungs. Specimens were taken from any doubtful lesions and microscopically examined.

### RESULTS

*Histology and pathogenicity of Vx7 and Vx2 carcinomas transplanted in domestic rabbits.*—Rous and his co-workers found Vx2 carcinomas to be more invasive than Vx7 when growing at intramuscular sites in the domestic rabbit (Rous, 1965). In our animals this was also observed. Many more metastases took place in the retroperitoneal lymph nodes and lungs in Vx2-bearing rabbits than in those carrying Vx7 tumors. The results of such observations on 180 animals which had been killed during the period of 60 days after inoculation of grafts of uniform size are summarized in Table II.

Histological sections of Vx2 and Vx7 taken from carcinomas in their 170th and 87th transplantation generations respectively are shown in Fig. 1 and 2. Histopathologically, the Vx7 still retains characteristics of squamous cell epidermal carcinoma whereas the Vx2 is a carcinoma of wholly anaplastic type.

*Induction of tumors in domestic rabbits with nucleic acid extracts from Vx7 carcinomas.*—After a rather prolonged incubation period of about 5 to 7 weeks, what appeared to be papillomatous growths arise at a number of the inoculation sites. These lesions later developed into typical papillomas (Fig. 3). In Table III, the results of tests on the tumorigenic activity of the preparations obtained from 8 independent extractions are summarized. The rate of positive " takes " was low, and ranged approximately from 14 to 7 per cent with an average of 9·1 per cent. It is noteworthy, however, that every nucleic acid preparation tested did give rise to several positive growths at least.

*Fate of the tumors induced with the nucleic acid extracts from Vx7 carcinomas.*—The majority of the tumors induced by the NA preparations from the carcinomatous tissues regressed after a certain period of growth on the skin of rabbits. A similar phenomenon of regression has been reported for both SP virus-induced (Evans, Gorman, Ito and Weiser, 1962) and NA-induced papillomas (Ito and Evans, 1961). The rate of regression of the papillomatous growths, however, was considerably higher among the Vx7 NA-induced tumors dealt with in the present study. In Fig. 8, the results of observation up to 30 weeks postinoculation, as concerns the fate of 61 Vx7 NA-induced papillomas in 23 rabbits are illustrated together with the results on 51 ordinary SP virus-induced papillomas in 17 rabbits. In the latter case, each animal received a standard inoculation of 0·2 ml. of SP virus ($10^{-1}$) at three sites in the back. The titer of the SP virus employed was $10^{-3}$ ($ID_{100}$).

The regression rate of both SP virus- and Vx7 NA-induced papillomas were about the same (33 per cent) and on the 10th week after inoculation. The rate of SP virus-induced tumors had become stabilized at a value of 47 per cent by the 12th week and remained unaltered up to 30 weeks, whereas the number of Vx7 NA-induced tumors regressing continued to increase gradually, *i.e.* 56 per cent at 15 weeks, 67 per cent at 20 weeks, 69 per cent at 25 weeks and finally 81 per cent at 30 weeks.

TABLE II.—*Macroscopic Metastases to Regional Lymph Nodes and Lungs in Rabbits Carrying Vx2 and Vx7 Carcinomas*

| | Vx2 carcinoma | | | | | Vx7 carcinoma | | | | |
|---|---|---|---|---|---|---|---|---|---|---|
| Days after transfer | No. of rabbits | Metastases in regional lymph nodes* | (Per cent incidence) | Metastases in lungs | (Per cent incidence) | No. of rabbits | Metastases in regional lymph node | (Per cent incidence) | Metastases in lungs | (Per cent incidence) |
| 0–20 | 5 | 1 | (20) | 0 | (0) | 2 | 0 | (0) | 0 | (0) |
| 21–30 | 41 | 24 | (59) | 10 | (49) | 6 | 1 | (17) | 0 | (0) |
| 31–40 | 62 | 53 | (85) | 36 | (58) | 9 | 5 | (56) | 2 | (22) |
| 41–50 | 25 | 21 | (84) | 14 | (56) | 15 | 9 | (60) | 3 | (20) |
| 51–60 | 10 | 10 | (100) | 5 | (20) | 5 | 3 | (60) | 0 | (0) |
| Total | 143 | 109 | (76) | 65 | (45) | 37 | 18 | (49) | 5 | (13) |

* The primary regional lymph nodes are in this case the retroperitoneal group.

TABLE III.—*Tumor-induction with Nucleic Acid Extracts from Transplanted Vx7 Carcinomas*

| Vx7 tumor: Extraction No. | Rabbit No.† | Generation | Weight (g.) | No. of animals used | No. growths per No. inoc. sites | Per cent take | Incubation period median and (range)* |
|---|---|---|---|---|---|---|---|
| 6 | N-965 | 81a | 121·1 | 7 | 7/84 | 8·3 | 33 (28–54) |
| 8 | N-967 | 81a | 239·5 | 8 | 9/96 | 9·4 | 46 (31–65) |
| 10 | N-969 | 81a | 150·2 | 6 | 7/72 | 9·7 | 36 (25–50) |
| 14 | N-973 | 81a | 90·5 | 8 | 12/84 | 14·3 | 45 (30–100) |
| 17 | N-979 | 81b | 83·7 | 7 | 6/84 | 7·1 | 40 (32–64) |
| 29 | N-991 | 81b | 50·1 | 7 | 6/84 | 7·1 | 45 (31–84) |
| 38 | N-1005 | 82 | 173·0 | 7 | 6/84 | 9·5 | 34 (29–72) |
| 40 | N-1007 | 82 | 137·8 | 7 | 6/84 | 7·1 | 35 (31–41) |
| | | | Total | 57 | 61/672 | 9·1 | |

* Days after inoculation of the NA extract.
† Number of the donor animal.

Among 9 papillomas on 5 rabbits which persisted over 30 weeks, 4 on two animals regressed between the 32nd and 37th week. Two other animals with 4 definite papillomas were accidentally killed after 39 and 43 weeks, respectively. The final papilloma in the last surviving rabbit showed a small ulcerated area at the base of growth on 55 week after inoculation. Microscopic observation of a biopsied specimen showed invasion of the dermis (Fig. 5 and 6), whereas the specimens from the unulcerated portion of the tumor still retained features of papillomatous growth (Fig. 4). The ulcerated area gradually expanded later, replacing the benign part of the tumor, and after 77 weeks the whole growth had ulcerated. The tumor now proved to be a squamous cell carcinoma (Rous and Beard, 1935) similar to those deriving from papillomas induced with the DNA derived from the SP virus (Ito, 1963).

*Test for detecting Shope papilloma virus in aqueous tissue extracts from Vx7 carcinomas.*—As shown in the work of Rous, tumorigenic activity of SP virus is sometimes demonstrable in Vx7 by employing aqueous tissue extracts and by carrying out the infectivity tests extensively (Rous, 1960). The aim of this work was to learn whether the Vx7 carcinomas used in the present study contained infectious SP virus. Ten per cent tissue extracts were prepared in PBS from four different tumors, all in their 81st generation. Intradermal injection and the puncture method (Ito and Evans, 1961) for inoculation of extracts were made in domestic rabbits, using a dose of 0·2 ml. for each site. Table IV depicts the results.

TABLE IV.—*Attempts to Detect " Intact Virus " in Aqueous Extracts of Vx7 Tissues*

| Donor rabbit No. | Tissue extract* (%) | No. of rabbits inoculated | No. growth per No. inoculation sites at 30 days | 60 days |
|---|---|---|---|---|
| N-967 | 10 | 9 | 0/108 | ±1†/108 |
| N-973 | 10 | 8 | 0/96 | 0/96 |
| N-979 | 10 | 9 | 0/108 | 0/108 |
| N-991 | 10 | 8 | 0/96 | 0/96 |
| | Total | 34 | 0/400 | ±1/400 |

* Prepared in PBS.
† A scanty growth appeared on 57 day but lasted only for 5 days and disappeared completely.

Only one small excrescence was seen in total of 400 sites of inoculation in 34 rabbits. The other sites failed to form growths during the 60 day period of observation.

*Attempts to induce tumors in domestic rabbits with nucleic acid extracts from Vx2 carcinomas.*—Efforts were made to induce papillomas in domestic rabbits with NA preparations extracted from Vx2 carcinomas by the same technic which proved to be effective in the case of Vx7s. Table V shows that no positive growth was visible at any of the 690 sites tested.

TABLE V.—*Attempts to Induce Tumor with Nucleic Acid Extracts from Transplanted Vx2 Carcinomas*

| Vx2 tumor | | | | | |
|---|---|---|---|---|---|
| Extraction No. | Rabbit No. | Generation | Weight (g.) | No. of rabbits inoculated | No. growth per No. inoculation sites |
| 30 | N-1119 | 167 | 93·7 | 7 | 0/84 |
| 31 | N-1121 | 167 | 132·9 | 8 | 0/96 |
| 32 | N-1125 | 167 | 219·8 | 7 | 0/84 |
| 33 | N-1117 | 167 | 107·5 | 6 | 0/66 |
| 34 | N-1118 | 167 | 143·7 | 8 | 0/96 |
| 35 | N-1124 | 167 | 116·3 | 7 | 0/96 |
| 36 | N-1120 | 167 | 109·5 | 7 | 0/80 |
| 37 | N-1123 | 167 | 125·6 | 7 | 0/88 |

### DISCUSSION

The present work shows that it is possible to produce papillomas in domestic rabbits with NA extracts whereas simple aqueous extracts from the same source only rarely induce such growths. These experimental results accord with the general view that Vx7 carcinomas still harbor some derivative of the Shope papilloma virus.

The Vx2 carcinoma was first transplanted in 1938 by Rous and his associates (Rogers, Kidd and Rous, 1960) and after 22 successive transfers during a period of 3½ years it was found still to immunize rabbits against SP virus. When tests for induced immunity were made again after 4 years, with rabbits bearing the carcinomas, none whatever was found. Despite what seemed to be favorable conditions

### EXPLANATION OF PLATES

All the sections were stained with hematoxylin and eosin.

FIG. 1.—Marginal section of a typical Vx2 carcinoma in its 170th transplantation generation. The tumor is completely anaplastic. × 160

FIG. 2.—Marginal section of a Vx7 carcinoma in its 87th transplantation generation. The features of squamous cell carcinoma are still preserved. At the left lower corner differentiation and keratinization can be seen. × 160

FIG. 3.—Keratinizing horn formed by an epidermal papilloma induced on a domestic rabbit by the inoculation of an NA extract from a Vx7 carcinoma. 17 weeks after inoculation. × 1·8

FIG. 4.—An ulcerated carcinomatous growth that had completely replaced a papilloma primarily induced by NA extract of a Vx7 carcinoma in its 81st transplantation generation. 77 weeks after inoculation. × 1·8

FIG. 5.—Vertical section through the palisaded midst of a Vx7 NA-induced papilloma. It is characteristic of an actively proliferating Shope papilloma. A small ulcerated area had recently appeared at its base. 55 weeks after inoculation. × 135

FIG. 6.—Vertical section through part of the ulcerated area illustrated in Fig. 4, showing carcinomatous cell invading the underlying dermis. × 240

FIG. 7.—Higher magnification of the portion of the field with carcinomatous cells shown in Fig. 5. Marked pleomorphism of the nuclei of the cells can be seen. × 600

Ito

Ito

5

6

7

FIG. 8.—The development and course of the 61 papillomas induced with Vx7 NA extracts in 23 domestic rabbits is shown together with that of 51 papillomas induced with Shope papilloma virus (SPV) in 17 rabbits. In Vx7 NA-induced tumors the incubation period is longer, there is much more tendency to regress and this occurs more rapidly, yet many of the growths persist.

in the present study, NA extracts from Vx2 carcinomas completely failed to yield papillomatous growth. These results seemingly accord with the documented fact that Vx2 carcinomas have lost the SP virus or its derivative at some time in the past (Rogers, Kidd and Rous, 1960). However, another conclusion is that the difference between Vx7 and Vx2 could be merely a quantitative one. Vx2 may also possess the active factor but only in amount below the sensitivity of the test with the NA extracts as employed in the present study. Findings presented in a preceding paper (Osato and Ito, 1967) suggest that such a possibility may indeed exist.

The author wishes to express his appreciation to Dr. Peyton Rous for the gift of Vx2 and Vx7 carcinoma strains and to Dr. C. A. Evans for valuable discussions. He is also indebted to Dr. S. Kuno, of Kanazawa University School of Medicine, for biochemical assay of the tissue extracts, to Dr. Y. Tubura, of Nara Medical College, for pathological examinations of the tissue specimens. This work was supported in part by grants from the National Cancer Institute (USPHS CA-08698) and from the Jane Coffin Child Memorial Fund for Medical Research.

### REFERENCES

DULBECCO, R. AND VOGT, M.—(1954) *J. exp. Med.*, **99**, 167.

EVANS, C. A., GORMAN, L. R., ITO, Y. AND WEISER, R. S.—(1962) *J. natn. Cancer Inst.*, **29**, 277.

ITO, Y.—(1960) *Virology*, **12**, 596.—(1962) *Cold Spring Harb. Symp. quant. Biol.*, **27**, 387.—(1963) *Acta Un. int. Cancr.*, **19**, 280.

ITO, Y. AND EVANS, C. A.—(1961) *J. exp. Med.*, **114**, 485.—(1965) *J. natn. Cancer Inst.*, **34**, 431.

OSATO, T. AND ITO, Y.—(1967) *J. exp. Med.*, **126**, 881.

ROGERS, S., KIDD, J. G. AND ROUS, P.—(1960) *Acta Un. int. Cancr.*, **16**, 129.

ROUS, P.—(1960) *Cancer Res.*, **20**, 765.—(1965) *Nature, Lond.*, **207**, 457.

ROUS, P. AND BEARD, J. G.—(1935) *J. exp. Med.*, **62**, 523.

ROUS, P., KIDD, J. G. AND SMITH, W. E.—(1952) *J. exp. Med.*, **96**, 159.